# Survival times of HIV/AIDS in different *AIDS Diagnostic and Treatment Guidelines* from 2006 to 2020 in Liuzhou, China

**DOI:** 10.1186/s12889-023-15662-3

**Published:** 2023-09-07

**Authors:** Susu Ke, Quan Fang, Jianguo Lan, Nini Qiao, Xinhong Zhang, Changping Xie, Yinguang Fan

**Affiliations:** 1 Department of Epidemiology and Biostatistics, School of Public Health, Anhui Medical University, Hefei, China; 2 Liuzhou Center for Disease Control and Prevention, 1 Tanzhongxi Road, Liuzhou, 545000 Guangxi Zhuang Autonomous Region China

**Keywords:** Antiretroviral therapy, Survival time, Cox regression model, People living with HIV/AIDS, Influencing factors

## Abstract

**Background:**

To compare the survival rates of four timing of treatment initiation for people living with HIV/AIDS provided in China in 2006, 2011, 2015, and 2018, and to investigate the factors impacting survival time.

**Methods:**

A people living with HIV/AIDS retrospective cohort study was in Liuzhou City from April 2006 to December 2020. The information was obtained from the National Comprehensive AIDS Prevention and Control Information System. Life tables and the Kaplan–Meier method were used to calculate participant survival rates and time. The univariate and multivariate Cox regression models were used to investigate the factors related to survival.

**Results:**

18,543 participants were included in this study. In four periods, the 1-year survival rates were 81%, 87%, 95%, and 95%. The 2-year survival rates were 76%, 85%, 93%, and 94%. The 3-year survival rates were 73%, 84%, 92%, and 94%. Results of multivariate Cox regression showed that sex, age of HIV diagnosis, ethnicity, household registration, occupation, marital status, the timing of treatment, education level, route of HIV transmission, whether receiving antiretroviral therapy (ART), and the count of CD4^+^T cells at baseline (count of CD4^+^T cells at HIV diagnosis) were factors that are significantly correlated with mortality caused by HIV infection.

**Conclusions:**

With the *Guidelines* updated from 2006 to 2020, the 1-, 2-, and 3-year survival rates of people living with HIV/AIDS in four periods tended to increase. The timing of treatment initiation of the updated edition of the *AIDS Diagnostic and Treatment Guidelines* (*Guidelines*) significantly prolonged the survival time of people living with HIV/AIDS.

**Supplementary Information:**

The online version contains supplementary material available at 10.1186/s12889-023-15662-3.

## Background

Acquired immune deficiency syndrome (AIDS), a major infectious disease, is caused by the human immunodeficiency virus (HIV).Which seriously affects the physical and mental health of humans [1]. In 2019, there were 1.7 million newly diagnosed HIV infections, 690,000 HIV-related deaths and 38 million people living with HIV/AIDS globally [2].

The development of antiretroviral therapy (ART) is one of the most significant achievements of modern medicine, slowing the progression of AIDS and reducing AIDS-related mortality [3, 4]. Since the "Four Free and One Care" policy was issued in China from 2003, Chinese government provided free antiretroviral medication to all people living with HIV/AIDS diagnosed. The "Four Free and One Care" policy includes: free ART, free voluntary counseling and testing, free schooling, and reduction of mother-to-child transmission. As a result, an increasing number of people living with HIV/AIDS are taking antiretroviral drugs [5].

ART has greatly improved the life expectancy and the quality of life for people living with HIV/AIDS [6, 7]. The best time to start ART in people living with HIV/AIDS varies between countries. In the latest *Guidelines* of America [8, 9], ART is advised for all newly infected individuals regardless their count of CD4^+^T cells at diagnosis. The World Health Organization (WHO) recommends that ART should be initiated when the count of CD4^+^T cells is less than 500 cells/mm^3^ in people living with HIV/AIDS [8]. In Europe, ART is recommended when the CD4^+^T cells count in RPWHA is less than 350 cells/mm^3^ and should be considered for individuals with CD4^+^T cells count of 350–500 cells/mm^3^ [10]. To date, four editions of the *AIDS Diagnostic and Treatment Guidelines* (*Guidelines*) have been issued in China, and the timing of starting ART treatment for people living with HIV/AIDS changed as the *Guidelines* were updated. In April 2006, China published the first edition of the *Guidelines*, ART is recommended when the CD4^+^T cells count is less than 200 cells/mm^3^ in HIV-positive asymptomatic people [11]. According to the second edition *Guidelines*, issued in October 2011, ART started when the counts of CD4^+^ T cells was less than 350 cells/mm^3^ [12]. The third edition *Guidelines*, issued in October 2015, recommended that ART should be initiated when the count of CD4^+^T cells is less than 500 cells/mm^3^ in people living with HIV/AIDS [13]. The latest *Guidelines*, published in December 2018, suggested no matter their count of CD4^+^T cells at baseline, all people living with HIV/AIDS should receive ART [14]. According to the publication dates of different editions of the *Guidelines*, the time from April 2006 to December 2020 was divided into four periods. Period one: April 2006-September 2011. Period two: October 2011-September 2015. Period three: October 2015-November 2018. Period four: December 2018-December 2020. It is critical to compare the survival times and factors related survival of people living with HIV/AIDS in different *Guidelines* in order to make clinical decisions and plan health service interventions [15].

HIV infection has created a significant global disease burden [16]. In China, there were 1.053 million people living with HIV/AIDS and 351,000 people died from HIV infection in 2020 [17]. Guangxi, located in the southern border region of China, has the second-largest population of people living with HIV/AIDS in South-Central China [18]. From 2010 to 2020, there were about 10,000 newly diagnosed HIV infections and 5,000 AIDS-related fatalities each year in Guangxi, ranking first in the morbidity and mortality rate of HIV in China [19, 20]. Liuzhou, in Guangxi Province, has the highest rate of HIV infection [21].

This study compared survival rates and investigated the influential factors of the people living with HIV/AIDS in different treatment indications, and provided evidence on prolonging the life span and improving the physical and mental health of the people living with HIV/AIDS.

## Methods

### Study populations

Participants were chosen based on the following factors: (1) Above 18 years old; (2) whose current address was "Liuzhou City, Guangxi Province" in the National Integrated AIDS Control Information System; (3) Confirmed HIV-infected or AIDS patient; (4) With a clear start date of antiviral treatment; (5) Deaths in the National Integrated AIDS Control Information System are AIDS-related deaths.

### Study design

A retrospective cohort study was conducted in Liuzhou. All information on HIV infectors aged 18 years old and above confirmed between 2006 and 2020 was extracted from National Integrated AIDS Control Information System. Data were reported to the Disease Control Department (CDC) by doctors with the consent of the patient. The CD4^+^ T cells count is the CD4^+^ T cells count at the time of the initial diagnosis, and for patients missing the initial CD4^+^ T cells count, we used the earliest primary CD4^+^ T cells count instead. The starting point of the study was the day participants were confirmed infected with HIV. The cohort follow-up deadline was December 31, 2020. The time from HIV diagnosis to death is defined as survival time, and the outcome variable was the death of the participant.

### Statistical analysis

For statistical analysis, IBM SPSS software, version 23.0, was utilized. Life tables and the Kaplan–Meier were used to calculated survival rates and the average survival time. Log-rank test was used to compare the survival rates of the four periods. The Mann–Whitney U test or the Chi-Square test for trend was performed to identify differences among the four groups. If univariate analysis revealed differences of more than 0.05, multivariate COX regression analysis was performed. *P* < 0.05 was used as the significance level.

## Results

### General demographic characteristics of people living with HIV/AIDS in Liuzhou City during different periods from 2006 to 2020

18,543 individuals were included in this study, 5,890(31.76%), 6,062(32.69%), 3,226(17.40%), and 3,365(18.15%) HIV infectors were confirmed in four periods, respectively. In this study, the majority of people living with HIV/AIDS were male (69.64%) and Chinese-Han (54.76%), nearly 57.64% of participants were aged 31–60, and heterosexual behavior is the principal route of HIV transmission (87.82%). From 2006 to 2020, the main household registration of people living with HIV/AIDS was Liunan District (46.35%), over half (56.96%) were married, and 58.94% of participants were farmers. The majority education level of them was Junior high school or less. In period one, the count of CD4^+^T cells of most patients was greater than 500/mm^3^ (22.99%). In period two and period four, many patients had the count of CD4^+^T cells of less than 200/ mm^3^ (24.30% and 36.20%, respectively). In period three, most of the patients had the count of CD4^+^T cells of 200–350/ mm^3^ (26.44%). Most patients in four periods received ART, accounting for 66.72%, 73.39%, 81.06%, and 77.12%, respectively. (Table 1).

**Table 1:** General demographic characteristics of people living with HIV/AIDS in Liuzhou City during different periods, 2006–2020

Covariate	2006–2020 (N%)	Period one (N%)	Period two (N%)	Period three (N%)	Period four (N%)	*P-value*
Sex						
Female	5,630 (30.36)	1,998 (33.92)	1,796 (29.63)	894 (27.71)	942 (27.99)	< 0.001
Male	12,913 (69.64)	3,892 (66.08)	4,266 (70.37)	2,332 (72.29)	2,423 (72.01)	
Age of diagnosis, y						
≤ 30	3,347 (18.05)	1,717 (29.15)	928 (15.31)	410 (12.71)	292 (8.68)	< 0.001
31 ~ 60	10,688 (57.64)	3,422 (58.10)	3,575 (58.97)	1,900 (58.90)	1,791 (53.22)	
≥ 61	4,508 (24.31)	751 (12.75)	1,559 (25.72)	916 (28.39)	1,282 (38.10)	
Household registration						
Liunan District	8,595 (46.40)	2,427 (41.20)	3,198 (52.80)	1,456 (45.10)	1,514 (45.00)	
Other districts in Liuzhou	8,074 (43.50)	2,621 (44.50)	2,356 (38.90)	1,493 (46.30)	1,604 (47.70)	< 0.001
Others	1,874 (10.10)	842 (14.30)	508 (8.40)	277 (8.60)	247 (7.30)	
Occupation						
Housekeeping	3,516 (18.96)	1,360 (23.09)	963 (15.89)	611 (19.94)	582 (17.30)	0.309
Farmer	10,928 (58.94)	2,957 (50.20)	3,922 (64.70)	1,911 (59.24)	2,138 (63.54)	
Laborer	778 (4.20)	299 (5.08)	225 (3.71)	162 (5.02)	92 (2.73)	
Business Service Provider	1,054 (5.68)	240 (4.07)	149 (2.46)	90 (2.79)	98 (2.90)	
Retirees	1,690 (9.11)	279 (4.74)	351 (5.79)	198 (6.14)	226 (6.72)	
Others	577 (3.11)	755 (12.82)	452 (7.46)	254 (7.87)	229 (6.81)	
Marital status						
Unmarried	4,350 (23.46)	1,468 (24.92)	3,330 (21.94)	775 (24.02)	777 (23.09)	< 0.001
Married	10,563 (56.96)	3,559 (60.42)	3,523 (58.12)	1,753 (54.34)	1,728 (51.35)	
Widowed/Divorced	3,630 (19.58)	863 (14.66)	1,209 (19.94)	698 (21.64)	860 (25.56)	
Ethnicity						
Han	10,153 (54.76)	3,422 (58.10)	3,398 (56.05)	1,667 (51.67)	1,666 (49.51)	< 0.001
Zhuang	7,169 (38.66)	2,223 (37.74)	2,292 (37.81)	1,287 (39.90)	1,367 (40.62)	
Others	1,221 (6.58)	245 (4.16)	372 (6.14)	272 (8.43)	332 (9.87)	
Educational level						
Primary or below	8,384 (45.21)	2,339 (39.71)	2,772 (45.73)	1,507 (46.71)	1,766 (52.48)	< 0.001
Junior high school	7,563 (40.79)	2,741 (46.54)	2,519 (41.55)	1,217 (37.72)	1,086 (32.27)	
Senior high school	1,854 (10.00)	665 (11.29)	559 (9.22)	319 (9.89)	311 (9.24)	
College or above	742 (4.00)	145 (2.46)	212 (3.50)	183 (5.68)	202 (6.01)	
Route of HIV transmission						
Heterosexual behavior	16,284 (87.82)	4,527 (76.86)	5,692 (93.90)	2,935 (90.98)	3,130 (93.02)	< 0.001
Homosexual behavior	523 (2.82)	29 (0.49)	115 (1.90)	194 (6.01)	185 (5.50)	
Injecting drug use	1,233 (6.65)	961 (16.32)	175 (2.89)	70 (2.17)	27 (0.80)	
Others	503 (2.71)	373 (6.33)	80 (1.32)	27 (0.84)	23 (0.68)	
Counts of CD4^+^ T cells (cells⁄mm ^3^)						
Not Tested	2,268 (12.23)	1,065 (18.08)	695 (11.46)	188 (5.83)	320 (9.51)	< 0.001
≤ 200	4,816 (25.97)	1,332 (22.61)	1,473 (24.30)	793 (24.58)	1,218 (36.20)	
200 ~ 350	4,081 (22.01)	1,103 (18.73)	1,325 (21.86)	853 (26.44)	800 (23.77)	
350 ~ 500	3,354 (18.09)	1,036 (17.59)	1,157 (19.09)	627 (19.44)	534 (15.87)	
> 500	4,024 (21.70)	1,354 (22.99)	1,412 (23.29)	765 (23.71)	493 (14.65)	
ART						
Yes	13,589 (73.28)	3,930 (66.72)	4,449 (73.39)	2,615 (81.06)	2,595 (77.12)	< 0.001
No	4,954 (26.72)	1,960 (33.28)	1,613 (26.61)	611 (18.94)	770 (22.88)	

Data outside of parentheses are the number of cases, and data in parentheses are the composition ratio (%)

### High-risk behaviors for HIV infection

With the *Guidelines* updated, the percentage of people having high-risk behavior changed. The proportion of patients having a history of injecting drugs tended to decline (Trend *χ* ^2^ = 911.272, *P* < 0.001). The percentage of patients with non-commercial sexuality has been increasing (Trend *χ* ^2^ = 3,855.978, *P* < 0.001). The rate of people living with HIV/AIDS with a history of commercial sexuality tended to increase (Trend *χ* ^2^ = 4,198.971, *P* < 0.001). The decreasing proportion of people living with HIV/AIDS whose mothers were HIV-positive (Trend *χ* ^2^ = 31.087, *P* < 0.001). Men who have sex with men (MSM) were becoming more prevalent. (Trend *χ* ^2^ = 285.489, *P* < 0.001). The rate of people living with HIV/AIDS who has a positive spouse/regular sex partner tended to decrease (Trend *χ* ^2^ = 53.615, *P* < 0.001). People living with HIV/AIDS with a history of surgery or blood donation showed a decreasing trend (Trend *χ* ^2^ = 42.881, *P* < 0.001). There was a decreasing trend in the proportion of patients with other exposure histories. (Trend *χ* ^2^ = 107.783, *P* < 0.001). (Table 2).

**Table 2:** Description of high-risk exposure history for people living with HIV/AIDS in Liuzhou City, 2006–2020

Covariate	Groupe				*χ* ^2^ test for trend	*P-value* for Trend
	period one (N%)	period two (N%)	period three (N%)	period four (N%)		
History of injecting drug use						
No	4,904 (83.26)	5,869 (96.82)	3,155 (97.80)	3,331 (98.99)	911.272	< 0.001
Yes	986 (16.74)	193 (3.18)	71 (2.20)	34 (1.01)		
History of non-commercial sexuality						
No	5,778 (98.10)	5,969 (98.47)	2,119 (65.69)	1,941 (57.68)	3,855.978	< 0.001
Yes	112 (1.90)	93 (1.53)	1,107 (34.31)	1,424 (42.32)		
History of commercial sexuality						
No	5,727 (97.23)	5,970 (98.48)	1,613 (50.00)	1,889 (56.14)	4,198.971	< 0.001
Yes	163 (2.77)	92 (1.52)	1,613 (50.00)	1,476 (43.86)		
Mother was HIV positive						
No	5,816 (98.74)	6,032 (99.51)	3,208 (99.44)	3,357 (99.76)	31.087	< 0.001
Yes	74 (1.26)	30 (0.49)	18 (0.56)	8 (0.24)		
MSM						
No	5,855 (99.41)	5,942 (98.02)	3,032 (93.99)	3,177 (94.41)	285.489	< 0.001
Yes	35 (0.59)	120 (1.98)	194 (6.01)	188 (5.59)		
Positive spouse/regular sex partner						
No	5,012 (85.09)	5,235 (86.36)	2,882 (89.34)	3,016 (89.63)	53.615	< 0.001
Yes	878 (14.91)	827 (13.64)	344 (10.66)	349 (10.37)		
History of surgery or blood donation						
No	5,808 (98.61)	6,022 (99.34)	3,213 (99.60)	3,357 (99.76)	42.881	< 0.001
Yes	82 (1.39)	40 (0.66)	13 (0.40)	8 (0.24)		
History of other exposures						
No	5,752 (97.66)	6,036 (99.57)	3,215 (99.70)	3,357 (99.76)	107.783	< 0.001
Yes	138 (2.34)	26 (0.43)	11 (0.34)	8 (0.24)		

### The survival rate of people living with HIV/AIDS in Liuzhou City from 2006 to 2020

**Fig. 1:**
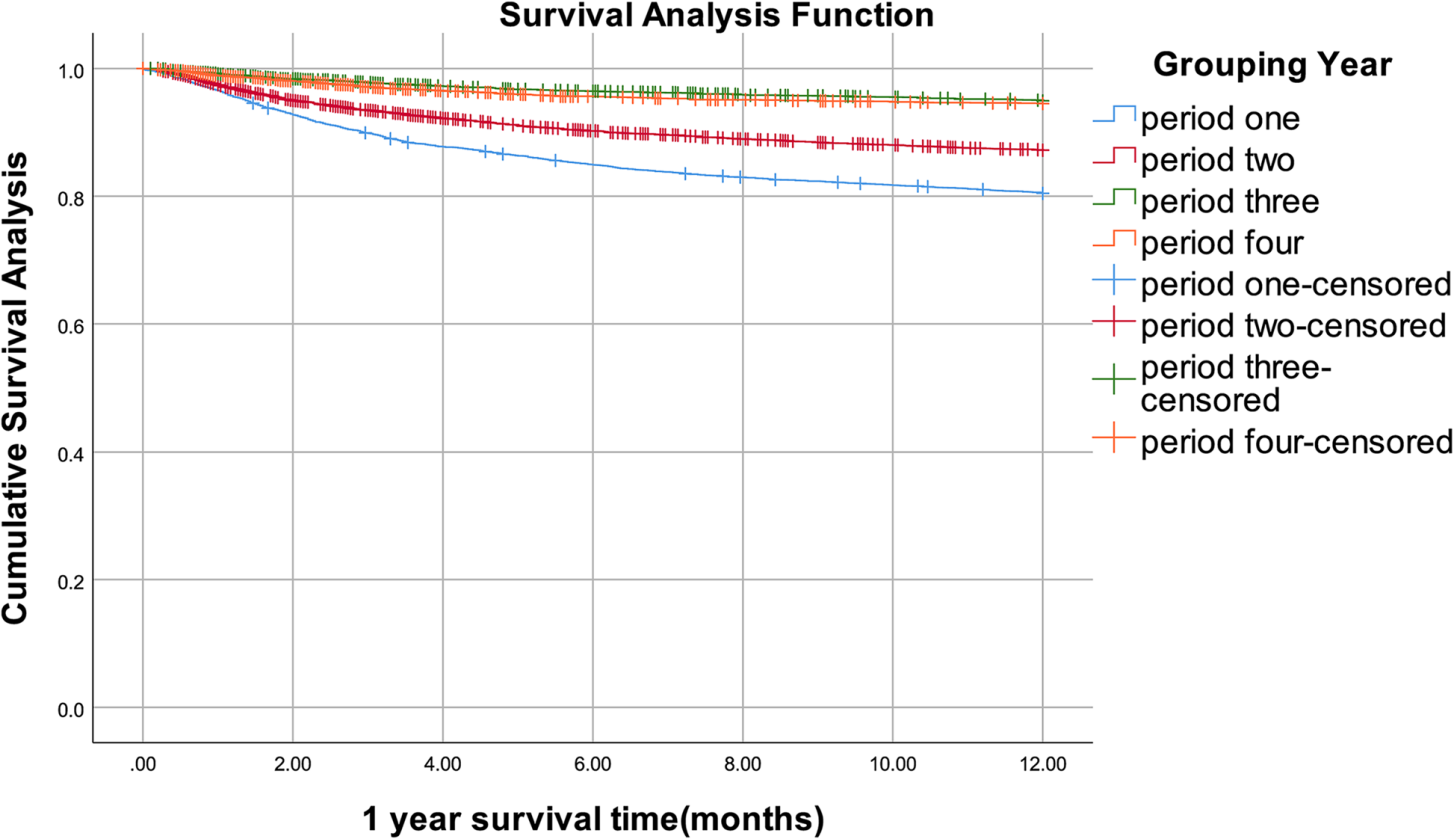
1-year survival curves of patients with people living with HIV/AIDS in Liuzhou City with different treatment options from 2006 to 2020

**Fig. 2:**
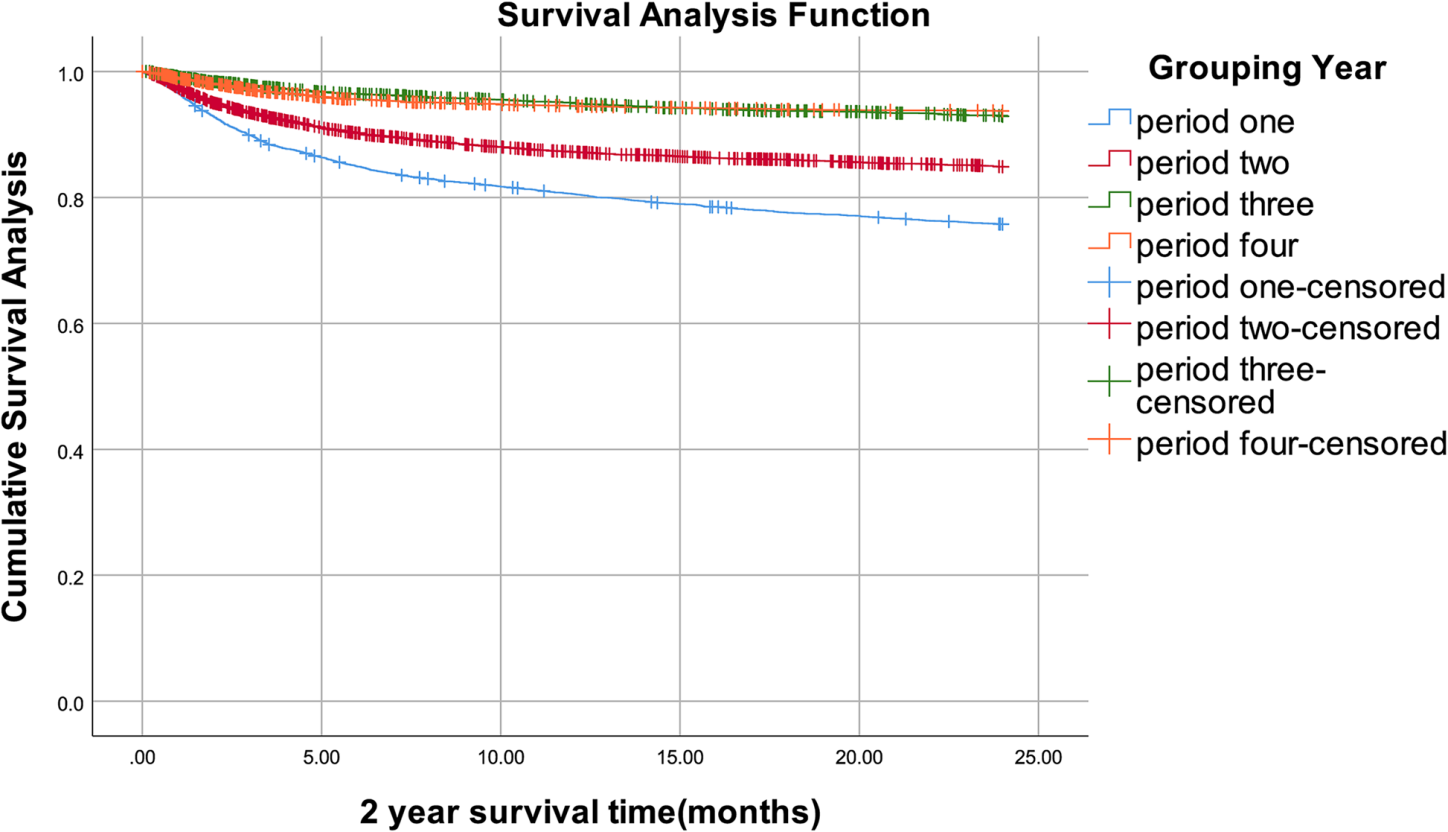
2-year survival curves of patients with people living with HIV/AIDS in Liuzhou City with different treatment options, 2006–2020

**Fig. 3:**
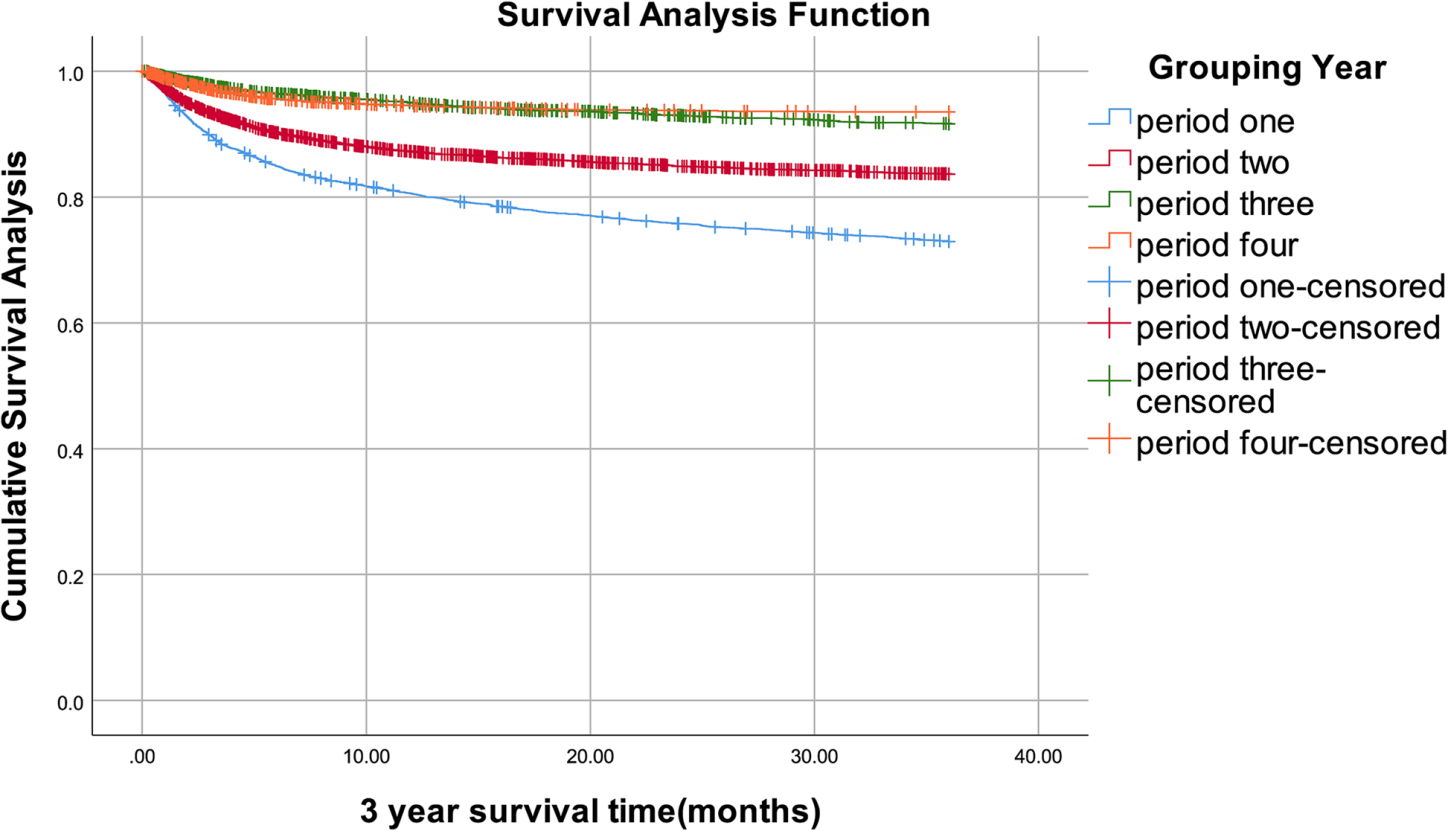
3-year survival curves for patients with people living with HIV/AIDS in Liuzhou City with different treatment options, 2006–2020

By December 31, 2020, of 18,543 people living with HIV/AIDS, 3,813 people had died. In four periods, 1-year survival rates were 81%, 87%, 95%, and 95%, respectively, 2-year survival rates were 76%, 85%, 93%, and 94%, 3-year survival rates were 73%, 84%, 92%, and 94%, respectively (Table 3). The survival curves demonstrated that the 1-, 2-, and 3-year survival rates all showed increasing trends. For infected patients with ART, in four periods, 1-year survival rates were 75%, 88%, 92%, and 98%, respectively, 2-year survival rates were 83%, 92%, 91%, and 100%, 3-year survival rates were 99%, 99%, 100%, and 100%, respectively. For infected patients no ART, in four periods, 1-year survival rates were 98%, 97%, 99%, and 98%, respectively, 2-year survival rates were 99%, 99%, 99%, and 99%, 3-year survival rates were 100%, 100%, 100%, and 100%, respectively (Supplementary Table 1). After log-rank test, the differences between groups were statistically significant in all four periods with ART, and also in no ART. (all *P* < 0.05). (Supplementary Table 2) The Kaplan–Meier method was used to assess the impact of four timing of treatment initiation on the people living with HIV/AIDS's survival time. In four periods, there were notable variations in 1-year survival rates. (*P* < 0.001). (Fig. 1). Similarly, the 2-year survival rates differed markedly between four periods. (*P* < 0.001). (Fig. 2). The 3-year survival rates in four periods varied dramatically. (*P* < 0.001). (Fig. 3). The timing of treatment for patients in period three and period four had a higher possibility of survival, suggesting the timing of treatment initiation of *Guidelines* (2018) and *Guidelines* (2015) significantly prolonged the survival time of patients.

**Table 3:** People living with HIV/AIDS survival rate in Liuzhou City, 2006–2020

Year	Time since diagnosis (months)	Number of Observers	Number of missed visits	Number of deaths	Mortality	Survival rate	Cumulative survival rate	Cumulative survival standard error
period one	0	5,890	17	1,143	0.19	0.81	0. 81	0.01
	12	4,730	14	281	0.06	0. 94	0.76	0.01
	24	4,435	16	162	0.04	0. 96	0.73	0.01
	36	4,257	3,752	505	0.21	0. 79	0.58	0.01
period two	0	6,062	296	755	0.13	0. 87	0.87	0
	12	5,011	172	134	0.03	0. 97	0.85	0
	24	4,705	145	65	0.01	0.99	0.84	0
	36	4,495	4,235	260	0.11	0.89	0.75	0.01
period three	0	3,226	267	153	0.05	0. 95	0.95	0
	12	2,806	99	59	0.02	0.98	0.93	0
	24	2,648	46	37	0.01	0.99	0.92	0.01
	36	2,565	2,515	50	0.04	0.96	0.88	0.01
period four	0	3,365	247	177	0.05	0.95	0.95	0
	12	2,941	34	25	0.01	0.99	0.94	0
	24	2,882	9	7	0.00	1.00	0.94	0
	36	2,866	2,866	0	0.00	1.00	0.94	0

### Factors influencing survival time

The Cox regression model was utilized to investigate the connection between baseline data and mortality. Factors influencing survival time in people living with HIV/AIDS were sex, age at diagnosis, household registration, the timing of treatment, occupation, marriage status, ethnicity, education level, route of transmission, whether receiving ART, and the count of CD4^+^T cells at baseline. Mortality risk tended to increase with increasing age at diagnosis. Among household registration, high mortality was seen in Liunan District. A lower degree of education was linked to a higher chance of dying. The survival time was significantly longer for patients in period four. Patients who didn't receive ART have a high mortality rate. Patients with a low count of CD4^+^ T cells have a higher mortality rate. (Table 4).

**Table 4:** Survival time of people living with HIV/AIDS in Liuzhou City, 2006–2020: Univariate and Multivariate Cox Regression

Covariate	Number of cases(N%)	Univariate cox regression		Multivariate cox regression	
		*P-value*	RR value (95% CI)	*P-value*	RR value (95% CI)
Sex					
Female	5,630 (30.36)		1.000		1.000
Male	12,913 (69.64)	< 0.001	1.965(1.815 ~ 2.1276)	< 0.001	1.239(1.138 ~ 1.348)
Age at diagnosis, y					
≤ 30	3,347 (18.05)		1.000		1.000
31 ~ 60	10,688 (57.64)	< 0.001	1.457(1.321 ~ 1.607)	< 0.001	1.306(1.175 ~ 1.452)
≥ 61	4,508 (24.31)	< 0.001	2.449(2.206 ~ 2.719)	< 0.001	1.583(1.387 ~ 1.808)
Occupation					
Housekeeping/housework/ standby jobs	1,360 (23.09)		1.000		
Farmer	2,957 (50.20)	< 0.001	1.239(1.135 ~ 1.352)	0.075	1.090(0.991 ~ 1.199)
Laborer	299 (5.08)	0.267	0.899(0.745 ~ 1.085)	0.157	1.148(0.948 ~ 1.391)
Business Service Provider	240 (4.07)	< 0.001	2.094(1.837 ~ 2.387)	0.027	1.180(1.019 ~ 1.366)
Retirees	279 (4.74)	0.085	1.121(0.984 ~ 1.278)	0.914	0.993(0.869 ~ 1.134)
Others	755 (12.82)	0.001	0.664(0.522 ~ 0.844)	0.687	0.951(0.744 ~ 1.215)
Household registration					
Liunan District	8,595 (46.40)		1.000		1.000
Other districts in Liuzhou	8,074 (43.50)	< 0.001	1.393(1.238 ~ 1.568)	< 0.001	1.461(1.293 ~ 1.650)
Others	1,874 (10.10)	< 0.001	1.256(1.115 ~ 1.415)	< 0.001	1.340(1.186 ~ 1.515)
Timing of treatment					
Period four	3,365 (18.15)				1.000
Period three	3,226 (17.40)	< 0.001	1.393(1.167 ~ 1.663)	< 0.001	2.334(1.954 ~ 2.789)
Period two	6,062 (32.69)	< 0.001	3.045(2.629 ~ 3.528)	< 0.001	3.701(3.191 ~ 4.294)
Period one	5,890 (31.76)	< 0.001	5.452(4.727 ~ 6.287)	< 0.001	5.388(4.649 ~ 6.244)
Marital status					
Unmarried	4,350 (23.46)		1.000		
Married	10,563 (56.96)	0.270	0.957(0.886 ~ 1.034)	0.932	1.004(0.920 ~ 1.096)
Widowed/Divorced	3,630 (19.58)	0.152	1.073(0.975 ~ 1.181)	0.867	0.965(0.867 ~ 1.074)
Ethnicity					
Han	10,153 (54.76)		1.000		1.000
Zhuang	7,169 (38.66)	< 0.001	0.815(0.762 ~ 0.871)	< 0.001	0.881(0.822 ~ 0.944)
Others	1,221 (6.58)	< 0.001	0.427(0.356 ~ 0.511)	< 0.001	0.642(0.535 ~ 0.771)
Educational level					
College or above	742 (4.00)		1.000		1.000
Junior high school	1,854 (10.00)	< 0.001	2.403(1.785 ~ 3.234)	0.019	1.410(1.059 ~ 1.876)
Senior high school	7,563 (40.79)	< 0.001	2.957(2.236 ~ 3.909)	0.092	1.277(0.961 ~ 1.698)
Primary or below	8,384 (45.21)	< 0.001	4.132 (3.129 ~ 5.456)	0.019	1.432(1.061 ~ 1.933)
Route of transmission					
Heterosexual	16,284 (87.82)		1.000		1.000
Homosexual	523 (2.82)	< 0.001	0.145(0.089 ~ 0.237)	0.006	0.496(0.300 ~ 0.819)
Injecting drug use	1,233 (6.65)	< 0.001	2.187(1.991 ~ 2.403)	0.109	0.915(0.822 ~ 1.020)
Others	503 (2.71)	< 0.001	2.448(2.126 ~ 2.819)	< 0.001	1.374(1.187 ~ 1.591)
ART					
Yes	13,589 (73.28)		1.000		1.000
No	4,954 (26.72)	< 0.001	15.815(14.689 ~ 17.027)	< 0.001	5.987(5.478 ~ 6.543)
Count of CD4^+^ T cells (cells⁄mm ^3^)					
Not Tested	2,268 (12.23)		1.000		1.000
≤ 200	4,816 (25.97)	< 0.001	0.013(0.011 ~ 0.016)	< 0.001	0.832(0.769 ~ 0.899)
200 ~ < 350	4,081 (22.01)	< 0.001	0.271(0.252 ~ 0.291)	< 0.001	0.197(0.173 ~ 0.225)
350 ~ < 500	3,354 (18.09)	< 0.001	0.046(0.041 ~ 0.051)	< 0.001	0.115(0.097 ~ 0.137)
> 500	4,024 (21.70)	< 0.001	0.024(0.028 ~ 0.020)	< 0.001	0.070(0.057 ~ 0.087)

The number of cases was partially missing in the multifactorial analysis

## Discussion

Patients in this study with the following characteristics constituted the majority of people living with HIV/AIDS in Liuzhou: male, 31–60 years old, farmer, married, primary school education or less, Han Chinese, heterosexual transmission, receiving ART, CD4^+^ T cell count below 200/mm^3^ at baseline, which is consistent with other studies [22, 23]. Notably, the count of CD4^+^T cells of most patients was less than 200/mm^3^, which could be owing to patients' late diagnosis. The count of CD4^+^T cells at baseline is critical for prognosis, as a low count of CD4^+^T cells at baseline may result in high mortality [24]. We found that the survival rate increased with the *Guidelines* updated, further suggesting the value of the current *Guidelines* is better than the previous one, in both ART and no-ART infected patients there were such results. There were many factors impacting on people living with HIV/AIDS's chances of survival. The risk of death was higher in males, consistent with the previous report [25]. Patients with the age of diagnosis of older than 30 years had a higher possibility of death. This may be due to the fact that as people get older, their immune systems weaken, making them more susceptible to disease and increasing their risk of dying. This is consistent with previous studies [26, 27]. Chinese-Han people died at a higher rate than other ethnic groupings. Higher-educated people living with HIV/AIDS had a lower mortality rate, which is in line with the results of studies in Yunnan Province [22].

We found the number of participants decreased by half from period two to three. Firstly, in our manuscript, the duration of period two was 48 months, and the duration of period three was 36 months, so the number of people in period three has been reduced. Secondly, in order to curb the development of AIDS, Guangxi Province implemented the "AIDS alleviation program" from 2010 to 2015 [28], especially the AIDS extended detection was done in the whole Province of Guangxi, so the number of new diagnoses was bigger in period two than period three, this period coincides with the period two in our manuscript. Finally, after expanded HIV testing, almost all existing HIV-infected patients have been diagnosed, so the number of newly diagnosed cases of HIV infection in period three has decreased.

In this manuscript, the CD4^+^ T cells count was the CD4^+^ T cells count at the time of HIV confirmed, it was not the CD4^+^ T cells count when infected persons start ART, and for participants who missing the initial CD4^+^ T cells count, the earliest primary CD4^+^ T cells count was used to replace it.

In this manuscript, the CD4^+^ T cells counts were not getting higher with the guidelines updated, which may related to the proportion of aged (≥ 61 years of age) HIV infected persons were increased in the four periods(trend*χ* ^*2*^ = 759.183, *P* < 0.001), An article pointed out that [29] the CD4^+^ T cells counts was lower in older HIV infected persons.

"Homosexual transmission" gave the lowest possibility of death in people living with HIV/AIDS. This may be ascribed to increased knowledge of HIV in MSM, they had more awareness of self-protection. The higher count of the CD4^+^T cells at baseline, the lower risk of death, which is consistent with the previous report [30]. People living with HIV/AIDS who didn't receive ART were at greater risk of death.

Studies [31–33] have shown that drugs such as methamphetamine and ketamine act on the central nervous system of the human body, producing strong euphoric effects that induce high-risk behavior and increase the spread of AIDS. According to this study, the proportion of patients with a history of intravenous drug use decreased from 2006 to 2020, indicating that HIV-related interventions in Liuzhou City are effective in recent years. However, it still cannot be ignored, and the publicity work on drugs and AIDS should be strengthened.

We observed a gradual increase in the survival rate of 18,543 people living with HIV/AIDS in four periods. According to the result of the univariate Cox regression model, whether receiving ART can impact the survival time of people living with HIV/AIDS, similar findings emerged with the multivariate Cox regression model. The timing of treatment also affected survival, which may be related to the increase in the count of CD4^+^T cells at baseline in the *Guidelines* for starting ART. In period one, only people whose count of CD4^+^T cells at baseline less than 200/mm^3^ were eligible for ART. Consistent with foreign studies [34, 35], individuals whose count of CD4^+^T cells at baseline was greater than 200/mm^3^ perished due to a failure to receive ART on time, this may account for the increased mortality rate [36]. The importance of early initiation of antiretroviral therapy is demonstrated.

In summary, the new *Guidelines* are better than previous ones. With the *Guidelines* updated from 2006 to 2020, the 1-, 2-, and 3-year survival rates tended to rise. Numerous elements impacted the people living with HIV/AIDS's survival time, and HIV education should be improved to raise public awareness and decrease the risk of HIV infection. Treatment initiated early, intensively, extensively, and continuously has the best potential to minimize mortality. This study further highlights the importance of early detection, diagnosis, and treatment. Therefore, HIV testing should be intensified so that an early diagnosis can be made.

There are some limitations to this study. Firstly, the data for this manuscript were extracted from National Integrated AIDS Control Information System, more information related to the mortality of HIV infected persons were not collected, such as adherence of ART, substance abuse and dietary habit. Secondly, Liuzhou has a special ethnic agglomeration, so it is difficult to extrapolate this result to other areas.

### Supplementary Information

**Additional file 1:** **SupplementaryTable 1.** Survival rates of people living with HIV/AIDSwith different treatment status in Liuzhou, 2006-2020. **Supplementary Table 2.** Comparisonof survival rates of infected patients in four periods with different treatmentstatus in Liuzhou, 2006-2020.

## Data Availability

The data that support the findings of this study are not openly available due to reasons of sensitivity and are available from the corresponding author upon reasonable request.

## Abbreviations

AIDS: Acquired immune deficiency syndrome
ART: Antiretroviral therapy
HIV: Human immunodeficiency virus
WHO: World Health Organization
