# Supplementary material for: Survival times of HIV/AIDS in different AIDS Diagnostic and Treatment Guidelines from 2006 to 2020 in Liuzhou, China
Source: BMC Public Health. 2023 Sep 7;23:1745. doi: 10.1186/s12889-023-15662-3 (PMC10483872; doi:10.1186/s12889-023-15662-3)
Supplement: Supplementary file 1 — Additional file 1: SupplementaryTable 1. Survival rates of people living with HIV/AIDSwith different treatment status in Liuzhou, 2006-2020. Supplementary Table 2. Comparisonof survival rates of infected patients in four periods with different treatmentstatus in Liuzhou, 2006-2020. [file 12889_2023_15662_MOESM1_ESM.docx]

Supplementary Table 1 Survival rates of people living with HIV/AIDS with different treatment status in Liuzhou, 2006-2020

| ART | Year | Time since diagnosis (months) | Number of Observers | Number of missed visits | Number of deaths | Mortality | Survival rate | Cumulative survival rate | Cumulative survival standard error |
| --- | --- | --- | --- | --- | --- | --- | --- | --- | --- |
| Yes | period one | 0 | 1960 | 17 | 1072 | 0.55 | 0.45 | 0.45 | 0.01 |
|  |  | 12 | 871 | 11 | 218 | 0.25 | 0.75 | 0.34 | 0.01 |
|  |  | 24 | 642 | 9 | 109 | 0.17 | 0.83 | 0.28 | 0.01 |
|  |  | 36 | 524 | 294 | 230 | 0.61 | 0.39 | 0.11 | 0.01 |
|  | period two | 0 | 1613 | 238 | 643 | 0.43 | 0.57 | 0.57 | 0.01 |
|  |  | 12 | 732 | 101 | 82 | 0.12 | 0.88 | 0.50 | 0.01 |
|  |  | 24 | 549 | 86 | 40 | 0.08 | 0.92 | 0.46 | 0.01 |
|  |  | 36 | 423 | 327 | 96 | 0.37 | 0.63 | 0.29 | 0.02 |
|  | period three | 0 | 611 | 204 | 115 | 0.23 | 0.77 | 0.77 | 0.02 |
|  |  | 12 | 292 | 48 | 21 | 0.08 | 0.92 | 0.71 | 0.02 |
|  |  | 24 | 223 | 21 | 19 | 0.09 | 0.91 | 0.65 | 0.02 |
|  |  | 36 | 183 | 171 | 12 | 0.12 | 0.88 | 0.57 | 0.03 |
|  | period four | 0 | 770 | 180 | 114 | 0.17 | 0.83 | 0.83 | 0.01 |
|  |  | 12 | 476 | 12 | 11 | 0.02 | 0.98 | 0.81 | 0.02 |
|  |  | 24 | 453 | 3 | 0 | 0 | 1.00 | 0.81 | 0.02 |
|  |  | 36 | 450 | 450 | 0 | 0 | 1.00 | 0.81 | 0.02 |
| No | period one | 0 | 3930 | 0 | 71 | 0.02 | 0.98 | 0.98 | 0 |
|  |  | 12 | 3859 | 3 | 63 | 0.02 | 0.98 | 0.97 | 0 |
|  |  | 24 | 3793 | 7 | 53 | 0.01 | 0.99 | 0.95 | 0 |
|  |  | 36 | 3733 | 3458 | 275 | 0.14 | 0.86 | 0.82 | 0.01 |
|  | period two | 0 | 4449 | 58 | 112 | 0.03 | 0.97 | 0.97 | 0 |
|  |  | 12 | 4279 | 71 | 52 | 0.01 | 0.99 | 0.96 | 0 |
|  |  | 24 | 4156 | 59 | 25 | 0.01 | 0.99 | 0.96 | 0 |
|  |  | 36 | 4072 | 3908 | 164 | 0.08 | 0.92 | 0.88 | 0.01 |
|  | period three | 0 | 2615 | 63 | 38 | 0.01 | 0.99 | 0.99 | 0 |
|  |  | 12 | 2514 | 51 | 38 | 0.02 | 0.98 | 0.97 | 0 |
|  |  | 24 | 2425 | 25 | 18 | 0.01 | 0.99 | 0.96 | 0 |
|  |  | 36 | 2382 | 2344 | 38 | 0.03 | 0.97 | 0.93 | 0.01 |
|  | period four | 0 | 2595 | 67 | 63 | 0.02 | 0.98 | 0.98 | 0 |
|  |  | 12 | 2465 | 22 | 14 | 0.01 | 0.99 | 0.97 | 0 |
|  |  | 24 | 2429 | 6 | 7 | 0 | 1.00 | 0.97 | 0 |
|  |  | 36 | 2416 | 2416 | 0 | 0 | 1.00 | 0.97 | 0 |

| ART |  | period one & period two | period one & period three | period one & period four | period two & period three | period two & period four | period three & period four |
| --- | --- | --- | --- | --- | --- | --- | --- |
| Yes | Log-rank*χ*^2^ | 20.365 | 38.543 | 46.822 | 17.962 | 31.068 | 9.457 |
|  | *P-value* | <0.001 | <0.001 | <0.001 | <0.001 | <0.001 | 0.002 |
| No | Log-rank*χ*^2^ | 106.605 | 226.733 | 496.943 | 77.584 | 237.859 | 34.279 |
|  | *P-value* | <0.001 | <0.001 | <0.001 | <0.001 | <0.001 | <0.001 |

Supplementary Table 2 Comparison of survival rates of infected patients in four periods with different treatment status in Liuzhou, 2006-2020
